# Contribution of 1p, 19q, 9p and 10q Automated Analysis by FISH to the Diagnosis and Prognosis of Oligodendroglial Tumors According to WHO 2016 Guidelines

**DOI:** 10.1371/journal.pone.0168728

**Published:** 2016-12-28

**Authors:** Karine Michaud, Marie de Tayrac, Myreille D’Astous, Céline Duval, Claudie Paquet, Oumar Samassekou, Peter Vincent Gould, Stéphan Saikali

**Affiliations:** 1 Department of Neurosurgery, Centre Hospitalier Universitaire de Québec, Québec, Canada; 2 Department of Genomic and Molecular Genetics, Centre Hospitalier Universitaire de Rennes, Rennes, France; 3 Department of Pathology, Centre Hospitalier Universitaire de Québec, Québec, Canada; National Cancer Institute, UNITED STATES

## Abstract

**Objective:**

To study the feasibility and the diagnostic and prognostic interest of automated analysis of 1p, 19q, 9p and 10q status by FISH technique in oligodendroglial tumors.

**Methods:**

We analyzed a retrospective series of 33 consecutive gliomas with oligodendroglial histology (originally diagnosed as 24 oligodendrogliomas and 9 oligoastrocytomas). For all cases, automated FISH analysis of 1p, 19q, 9p and 10q status were performed and compared to clinical and histological data, ATRX, IDH1R132H and alpha-internexin status (studied by immunohistochemistry) and overall survival (OS). Manual analysis of 9p and 10q status were also performed and compared to automated analysis to verify the concordance of the two methods.

**Results:**

The 33 gliomas were reclassified into 13 low-grade oligodendrogliomas (OII), 10 anaplastic oligodendrogliomas (OIII), 3 diffuse astrocytomas (AII), 3 anaplastic astrocytomas (AIII) and 4 glioblastomas (GBM) according to the WHO 2016 histological criteria. The 1p and/or 19q imbalanced status were restricted to astrocytomas with no correlation to their grade or their OS. Chromosome 9p deletion was restricted to OIII (70%) and GBM (100%) and was correlated with a shorter OS in the total cohort (p = 0.0007), the oligodendroglioma cohort (p = 0.03) and the astrocytoma cohort (p = 0.001). Concordance between 9p manual and automated analysis was satisfactory (81%, κ = 0.69). Chromosome 10q deletion was restricted to GBMs (50%) and was correlated with a poor OS in both the total cohort (p = 0.003) and the astrocytoma (AS) cohort (p = 0.04). Concordance between manual and automated analysis was satisfactory (79%, κ = 0.62).

**Conclusion:**

Automated analysis of 1p, 19q, 9p and 10q status by FISH is a reliable technique which allows for refined classification of oligodendroglial tumors. 1p and/or 19q imbalanced status is evidence of astrocytic differentiation. 9p deletion is found in high grade oligodendrogliomas and astrocytomas with a poor OS. 10q is related to GBM status and a poor OS.

## Introduction

In the 2016 WHO Classification of Tumours of the Central Nervous System, determination of chromosome 1p and 19q status is a core criterion in the diagnosis of oligodendroglial tumors. Oligodendrogliomas (OGs) are henceforth defined by the molecular association of 1p/19q whole arm codeletion and IDH1/2 mutation, other configurations being considered as astrocytic neoplasms or low-grade neuroepithelial tumors (LGNTs) [1–3]. Codeletion of 1p and 19q also guides the therapeutic management of these tumors since it has been associated with sensitivity to chemotherapy and improved outcome as well as increased benefit of adjuvant chemotherapy given after radiotherapy [4–7].

Other chromosome aberrations which have been described in association with oligodendroglial tumors include 9p loss, 9q loss, 10q loss, 11q gain, whole chromosome 7 gain and whole chromosome 4 loss [8–11], but their prognostic significance is less clear.

In our institution, the molecular study of chromosome 1p and 19q status is performed by FISH technique on paraffin embedded tissue and the results are routinely classified by automated analysis with a high concordance as compared to manual analysis [12]. In order to broaden our panel of molecular cytogenetic analyses for gliomas in general and for oligodendroglial tumors in particular, we decided to document the status of chromosome arms 9p and 10q by FISH technique using commercial probes and to assess whether automated analysis, as is done already for 1p and 19q, would be feasible. In order to validate the effectiveness of our FISH technique and its diagnostic and prognostic value, manual and automated analysis of 9p and 10q status was performed on a retrospective series of 33 consecutive oligodendroglial tumors operated in our institution, originally diagnosed as an OG or an oligoastrocytoma (OA) with a WHO grade of II or III. In a first step the cohort was reclassified according to the WHO 2016 criteria [1] which takes into account both classical histological criteria such as mitotic index, microvascular proliferation (MVP) and necrosis, and newer molecular criteria such as isocitrate dehydrogenase (IDH1/2) gene mutation status (typically studied by immunohistochemistry [1], and 1p/19q chromosome arms deletion status (studied in our case by automated FISH). IDH1/2 status is necessary in order to provide an integrated diagnosis of diffuse gliomas. We also looked for alpha-thalassemia/mental retardation (ATRX) gene mutation status, since ATRX loss by immunohistochemistry is characteristic, but not required for diagnosis of diffuse astrocytoma, IDH-mutant by the WHO. Alpha-internexin (INA) protein has been described as a surrogate marker of 1p/19q co-deletion [13] and was also included in our immunohistochemical panel along with MIB-1/Ki-67. Expression of 9p and 10q status was then studied by FISH using both manual and automated analysis using our internal algorithm as previously established for 1p and 19q analysis [12]. Results were compared to determine their concordance level. 9p and 10q was compared to histological diagnostic and grade to determine their diagnostic value and compared to our cohort overall survival (OS) to study their prognostic value. To further clarify the place of 1p and/or 19q imbalanced status in the definition and the management of oligodendroglial tumors and their mimics as described in our previous work [12], and that of others [14,15] we studied the correlation of 1p and 19q imbalanced status with IDH1, ATRX and 1p/19q codeletion status as well as to OS in our reclassified cohort.

## Materials and Methods

### Ethics statement

The Research Ethics Committee of the Centre Hospitalier Universitaire de Québec was consulted for this study and declared that its approval was not necessary. The committee waived the need for consent, the aim of this study being the optimization of our institution diagnostic tools with anonymized data (notice 2016–2638): S1 File. Tumor samples were collected and anonymized by the Pathology Service of the Centre Hospitalier Universitaire de Québec (Hôpital de l’Enfant-Jésus, Quebec City, Canada).

### Patients and tissue specimens

Formalin fixed paraffin-embedded (FFPE) tissue from 33 consecutive oligodendroglial brain tumor samples (biopsies or surgical resections) studied in our institution between 1998 and 2004 were selected for this study. These cases, initially diagnosed as 14 OII, 10 OIII, 4 OAII and 5 OAIII, were re-analyzed by two neuropathologists (PVG and SS) and reclassified according to the WHO 2016 guidelines [1] into 13 OII, 10 OIII, 3 AII, 3 AIII and 4 GBM.

### Immunohistochemistry

Immunohistochemistry (IHC) was performed on 4 μm thick FFPE sections with the EnVision^TM^ FLEX+ detection system on Dako Autostainer 48 (Dako, Mississauga, Ontario). Reactions were visualized by EnVision^TM^ FLEX DAB+ Chromogen (Dako, Glostrup, Denmark).

The cases were stained with monoclonal antibodies IDH1R132H (clone H09; 1/50; Optistain), internexin alpha (INA; clone 2E3; 1/200; Santa Cruz), ATRX (clone D-5; 1/50; Santa Cruz) and Ki67 (clone Mib1; no dilution; Dako) with an incubation of 30 min for each.

Ki67 expression was scored as percentage by counting the immunostained nuclei of 100 neoplastic cells in the most positive area. INA expression was scored as positive (if ≥ 10% of cells were positive with at least one cluster of positive cells) or negative [13]. IDH132H expression was scored as positive (mutated) if at least one positive cell was observed or negative (wild type). ATRX expression was scored as positive (wild type) if ≥ 10% of cells were positive or negative (mutated) [16].

### FISH technique

FISH analysis was performed using commercial probes for chromosomes 1p36.32 (TPRG1L), 1q25.2 (ABL2), 9p21.3 (CDKN2A), 9q34.12 (ABL DF), 10p12.31 (MLLT10), 10q23.31 (PTEN), 19p13.2 (ZNF443), and 19q13.33 (GLTSCR2) purchased from Agilent Technologies (SureFISH; Mississauga, ON). Targeted chromosomal arms were labelled by a red fluorophore (namely 1p36.32, 9p21.3, 10q23.31 and 19q13.33). The opposite chromosomal arm served as control and was labelled in green.

5-μm-thick formalin-fixed, paraffin-embedded sections were deparaffinized, immersed in HCL 0.2 N, pretreated in a sodium citrate solution (pH 6.0) and digested in pepsin solution. For each chromosome, 5 μl of each chromosomal arm probe were added to 150 μl of tDenHyb-1 solutions (Insitus Biotechnologies, Albuquerque, NM, USA) and mixed with the corresponding opposite arm (namely 1p36 with 1q25, 9p21 with 9q34, 10q23 with 10p12 and 19q13 with 19p13). Target DNA and probes were codenatured at 74°C for 5 minutes and incubated at 37°C overnight in a humidified hybridization chamber (ThermoBrite, Abbott Molecular Inc.). Posthybridization washes were performed in NP40 0.3%/2×SSC (pH 7) at 75°C for 2 minutes. Finally, the slides were air dried and counterstained with DAPI (4′,6-diamidino-2-phenylindole) diluted in Vectashield (Vector, Burlingame, CA, USA).

Signal acquisition was performed for each slide over 12 more representative areas. These areas were automatically captured at x400 using a Metasystem station (Zeiss MetaSystems, Thornwood, NY) equipped with a Zeiss Axioplan fluorescent microscope. The acquired images were then used as the basis for the manual and the automated counting assays. Automated analysis was performed using the Metafer 4 software (Metasystems).

### FISH interpretation

FISH manual analysis was performed by a single observer (SS) on 100 non-overlapping nuclei for green ‘G’ (control) and red ‘R’ (target) signals. Automatic analysis was performed on all tumor cells identified by the Metafer 4 software using our internal algorithm [12]. This sampled a mean of 744 cells (min: 289 –max: 1453 –median: 735) for chromosome 1, 815 cells (min: 81 –max: 1371 –median: 843) for chromosome 9, 649 cells (min: 45 –max: 1423 –median: 681) for chromosome 10 and 701 cells (min: 347 –max: 1482 –median: 684) for chromosome 19 respectively.

For both 1p and 19q, the cut off was set at 55% for a diagnosis of chromosome deletion and 20% for imbalanced chromosome status, as described in our previous paper. For 9p and 10q, the cut-off was calculated on a series of 5 non-neoplastic brain tissue samples including epilepsy surgery cases and normal autopsy brains, using mean +3 SD and was set at 30% and 25% respectively for chromosome deletion status and 40% and 55% respectively for imbalanced chromosome status. A tumor was classified as deleted for one of these chromosomes if the percentage (%) of deleted nuclei exceeded the deletion status cut off. In the other cases a tumor was classified as (1) normal chromosome status if the percentage of deleted plus imbalanced nuclei was less than the deletion cut-off or (2) imbalanced if the sum of imbalanced + deleted nuclei was greater than or equal to the deletion cut-off [17,18]. Cases exceeding the imbalanced status cut off were considered imbalanced independently of their deletion status.

Since some studies [8,10] have suggested that loss of 9q or 10p –which served as control arms in our series–may occur in oligodendroglial tumors, we decided to include the additional combination 1R/1G in our algorithm for these 2 chromosomes. Cases displaying a rate of 1R/1G greater than or equal to their respective deletion cut-off (namely 30% and 25% respectively) were considered as deleted for 9q or 10p.

### Statistical analyses

All statistical analyses were carried out with the R statistical environment (http://www.R-project.org/). Concordance between manual analysis and automated analysis was estimated by calculating Cohen's kappa coefficient (κ) with the Kappa function of the R package *vcd*. We considered that a κ value between 0.6 and 0.8 reflects good agreement and that a value > 0.8 constitutes high concordance. Chi-square test was performed for group comparisons between clinical, histological and molecular status data, and p values < 0.05 were considered as significant. In order to identify histological and/or molecular data related to overall survival, survival curves were obtained according to the Kaplan-Meier method and compared using the log-rank test. Cohort follow up was recorded until 2016/03/01. The threshold for statistical significance was set at p < 0.05. The following variables were searched for prognostic significance in the whole group: age at diagnosis (cut off = 50 years), sex, microvascular proliferation (MVP), number of mitoses (cut off = 5, median), histological grade, Ki67 labelling index (cut off = 12%), INA expression (cut off = 10%), IDH mutation (present/absent), ATRX mutation (cut off = 10%), 1p deletion or imbalance, 9p deletion or imbalance, 9q deletion, 10q deletion or imbalance, 19q deletion or imbalance. Factors that were significant in univariate analysis were entered as candidate variables in the multivariate Cox proportional hazard regression model analysis.

## Results

### Clinical data

Clinical data are summarized in Table 1. Our series of 33 patients included 18 males (55%) and 15 (45%) females. Patients underwent open surgery with gross total or subtotal tumor resection (30/33 = 91%) or stereotactic biopsies (3/33 = 9%). For statistical analysis purpose the tumor location was assigned to be the brain’s lobe within which the largest volume of the tumor resided, even if several lobes were affected. In our series the majority of cases was located in the frontal lobe (23/33 = 70%) followed by the temporal lobe (7/33 = 21%) and the parietal lobe (3/33 = 9%). Seven cases among the 33 cases were recurrent tumors (21%). 7 cases had no post-operative treatment (21%), 5 cases received post-operative radiotherapy alone with a mean total dose of 50Gy (15%), 11 cases received chemotherapy alone with PCV or Temozolomide (34%) and 10 were treated with adjuvant PCV or Temozolomide radiochemotherapy (30%). The cohort mean OS was of 94 months (min = 1; max = 211 and median = 87). Seven patients were still alive (21%) at the end of the study while and 26 were deceased (79%).

**Table 1:** Clinical, histological and molecular data of the whole series according to the tumoral type and grade.

Histological Type		Total cohort	OII	OIII	AII	AIII	GBM	OII/ OIII	O/A	AII/AIII+GBM	AII+AIII / GBM
**N**		33	13	10	3	3	4				
**Recurrence**	**Yes (%)**	7(21)	1(8)	2 (20)	1 (33)	3 (100)	0 (0)	NS	NS	NS	**0,02**
	**No (%)**	26 (79)	12 (92)	8 (80)	2 (67)	0 (0)	0 (0)				
**Age at diagnosis**	**Mean**	44	45	47	38	39	44	NS	NS	NS	NS
	**Median**	42	42	44	38	35	43				
**Sex**	**Male (%)**	18 (55)	8 (62)	6 (60)	0 (0)	2 (67)	2 (50)	NS	NS	**0,03**	NS
	**Female (%)**	15 (45)	5 (38)	4 (40)	3 (100)	1 (33)	2 (50)	NS	NS	NS	NS
**Extent of surgery**	**Biopsy (%)**	3 (9)	2 (15)	0 (0)	0 (0)	0 (0)	1 (25)	NS	NS	NS	NS
	**Surgery (%)**	30 (91)	11 (85)	10 (100)	3 (100)	3 (100)	3 (75)	NS	NS	NS	NS
**Localization**	**Frontal (%)**	23 (70)	10 (77)	6 (60)	3 (100)	2 (67)	2 (50)	NS	NS	NS	NS
	**Temporal (%)**	7 (21)	3 (23)	2 (20)	0 (0)	0 (0)	2 (50)	NS	NS	NS	NS
	**Parietal (%)**	3 (9)	0 (0)	2 (20)	0 (0)	1 (33)	0 (0)	NS	NS	NS	NS
**Postoperative treatment (%)**	**None**	7 (21)	3 (23)	3 (30)	0 (0)	0 (0)	1 (25)	NS	NS	NS	NS
	**Radiotherapy**	5 (15)	1 (8)	2 (20)	2 (67)	0 (0)	0 (0)	NS	NS	**0,01**	NS
	**Chemotherapy**	11 (34)	4 (31)	3 (30)	0 (0)	1 (33)	3 (75)	NS	NS	**0,03**	NS
	**Radio + chemotherapy**	10 (30)	5 (38)	2 (20)	1 (33)	2 (67)	0 (0)	NS	NS	NS	NS
**Status (%)**	**Alive**	7 (21)	4 (31)	2 (20)	1 (33)	0 (0)	0 (0)	NS	NS	NS	NS
	**Dead**	26 (79)	9 (69)	8 (80)	2 (67)	3 (100)	4 (100)				
**MVP(%)**	**Endocrinoid**	18 (55)	13 (100)	0 (0)	3 (100)	3 (100)	0 (0)	**<0,0001**	NS	NS	**0,05**
	**Glomeruloid**	15 (45)	0 (0)	10 (100)	0 (0)	0 (0)	4 (100)	NS	NS	NS	0,05
**Calcifications (%)**		12 (36)	5 (38)	5 (50)	0 (0)	2 (67)	0 (0)	NS	NS	NS	**0,001**
**Mitoses / 10 HPF**	**Mean**	3,6	1,7	7	0	3,7	4,3	**0,01**	NS	**0,001**	NS
	**Median**	3	2	6	0	3	5				
**Mib1 positive (%)**	**Mean**	14,6	10	21	8	8	23	**0,01**	NS	NS	**0,01**
	**Median**	12	10	21	8	8	20				
**INA**	**positive (%)**	20 (61)	11 (85)	8 (80)	0 (0)	0 (0)	1 (25)	NS	**<0,0001**	NS	NS
**IDH 132H**	**positive (%)**	29 (88)	12 (92)	10 (100)	3 (100)	3 (100)	1 (25)	NS	**0,04**	NS	**0,05**
**ATRX**	**positive (%)**	29 (88)	13 (100)	10 (100)	2 (67)	1 (33)	3 (75)	NS	**0,0006**	NS	NS
**Chr 1p**	**loss (%)**	26 (79)	13 (100)	10 (100)	1 (33)	1 (33)	1 (25)	NS	**<0,0001**	NS	NS
	**no deletion (%)**	3 (9)	0 (0)	0 (0)	0 (0)	1 (33)	2 (50)	-	**0,05**	NS	NS
	**imbalance (%)**	4 (12)	0 (0)	0 (0)	2 (67)	1 (33)	1 (25)	-	**0,01**	NS	NS
**Chr 19q**	**loss (%)**	23 (70)	13 (100)	10 (100)	0 (0)	0 (0)	0 (0)	NS	**<0,0001**	NS	NS
	**no deletion (%)**	5 (15)	0 (0)	0 (0)	0 (0)	2 (67)	3 (75)	-	**0,03**	**0,03**	NS
	**imbalance (%)**	5 (15)	0 (0)	0 (0)	3 (100)	1 (33)	1 (25)	-	**0,03**	**0,03**	NS
**Chr 9p**	**loss (%)**	11 (33)	0 (0)	7 (70)	0 (0)	0 (0)	4 (100)	**0,002**	NS	NS	**0,001**
	**no deletion (%)**	17 (52)	11 (85)	2 (20)	2 (67)	2 (67)	0 (0)	**0,02**	NS	NS	**0,02**
	**imbalance (%)**	5 (15)	2 (15)	1 (10)	1 (33)	1 (33)	0 (0)	NS	NS	NS	NS
**Chr 9q**	**loss (%)**	2 (6)	0 (0)	2 (20)	0 (0)	0 (0)	0 (0)	NS	NS	NS	NS
**Chr 10q**	**loss (%)**	2 (6)	0 (0)	0 (0)	0 (0)	0 (0)	2 (50)	-	NS	NS	**0,06**
	**no deletion (%)**	27 (82)	11 (85)	9 (90)	2 (67)	3 (100)	2 (50)	NS	NS	NS	NS
	**imbalance (%)**	4 (12)	2 (15)	1 (10)	1 (33)	0 (0)	0 (0)	NS	NS	NS	NS
**Chr arm alteration**	**Mean**	2,6	2,3	2,9	2,7	1,7	2,25	NS	NS	NS	NS
**Chr arm deletion**	**Mean**	1,9	2	2,7	0,3	0,3	1,75	0,001	<0,0001	NS	0,004

Statistically significant: p-values <0.05, NS: non significant,—: Not applicable, MVP: microvascular proliferation, INA: alpha-internexin, IDH: isocitrate dehydrogenase, ATRX: Alpha Thalassemia Mental Retardation, Chr: chromosome, HPF: high power-field, OII: grade II oligodendroglioma, OIII: anaplastic oligodendroglioma, AII: diffuse astrocytoma, AIII: anaplastic astrocytoma, GBM: glioblastoma

### Histological data

After histological review using WHO 2016 criteria, 2 cases initially diagnosed as OII were reclassified as AII and 2 cases initially diagnosed as OIII were reclassified as GBM. The 4 cases initially diagnosed as OAII were reclassified as 1 OII, 1 AII, 1 AIII and 1 GBM. The 5 cases initially diagnosed as OAIII were reclassified as 2 OIII, 2 AIII and 1 GBM. No significant difference was observed between the age, the localization and the postoperative treatment distribution according to the final histological subtypes (Table 1). The AII subgroup showed an exclusive female distribution unlike the AIII and GBM subgroups (p = 0.03). MVP as defined in the WHO 2016 classification was present only in the OIII and GBM subgroups and absent from the OII, AII and AIII subgroups (p<0.0001). Calcifications were mainly observed in OGs (10/23 = 44%) and occasional ASs (2/10 = 20%) but not in GBMs (p = 0.001). Mitoses were more numerous in high grade oligodendrogliomas and astrocytomas than in their low grade counterparts (p = 0.01 and 0.001 respectively).

### Immunohistochemistry

INA expression was present in the majority of OII and OIII (85% and 80%) respectively, one astrocytoma and one GBM (Table 1). This distribution, although not exclusive to OGs, was highly correlated with membership in the OG cohort (p<0.0001).

IDH1R132H expression was present in the majority of our cases: 92% of OII, 100% of OIII, 100% of AII, 100% of AIII and 25% of GBM with a significant predominance in the OG cohort than in the astrocytoma (AS) cohort (p = 0.04). Within the AS cohort, IDH1R132H expression in AII + AIII cohort was statistically significant when compared to GBMs (p = 0.05): Table 1. The only IDH1R132H negative tumour which otherwise fulfilled the WHO 2016 criteria for OII was mutated for IDH2 on complementary molecular analysis (data not shown).

The lack of ATRX protein expression, which is a surrogate marker for ATRX gene mutation [16], was observed in 4/10 AS (40%): 1/3 AII, 2/3 AIII and 1/4 GBM (33%, 67% and 25% respectively). No loss of ATRX protein expression was observed in tumours which met the WHO 2016 criteria for OG. The difference in ATRX expression between OGs and ASs was statistically significant (p = 0.0006): Table 1.

### Molecular data

#### 1p/19q FISH analysis

Automated analysis of 1p and 19q status was performed by the Metafer analysis software using our institutional algorithm [12]. 5 cases stained for 1p and 4 cases stained for 19q had weak fluorescent signals which led to a high rate of rejected cells by automated analysis, requiring a secondary manual counting. As defined by the 2016 WHO criteria, all adult OGs are 1p and 19q codeleted (Table 1) and this codeletion appeared strongly correlated to the OG cohort (p<0.0001). In our series, 1p deletion was also observed in 3 AS (1/3 AII, 1/3 AIII and 1/4 GBM). In contrast, all tumors with normal and/or imbalanced status for 1p and 19q were part of the AS cohort. The distribution of chromosome anomalies for 1p and 19q in AS as compared to OGs was statistically significant (p = 0.01 for 1p and p = 0.03 for 19q) (Table 1). 1p and/or 19q imbalanced status was observed in the majority of AII and some AIII and GBM. 1p imbalanced status was observed in 4 ASs (2/3 AII, 1/3 AIII and 1/4 GBM). 19q imbalanced status was observed in 5 ASs (3/3 AII, 1/3 AIII and 1/4 GBM). No cases in the OGs cohort showed 1p and/or 19q imbalanced status concomitant with the codeletion status.

#### 9p/9q FISH analysis

**Fig 1:**
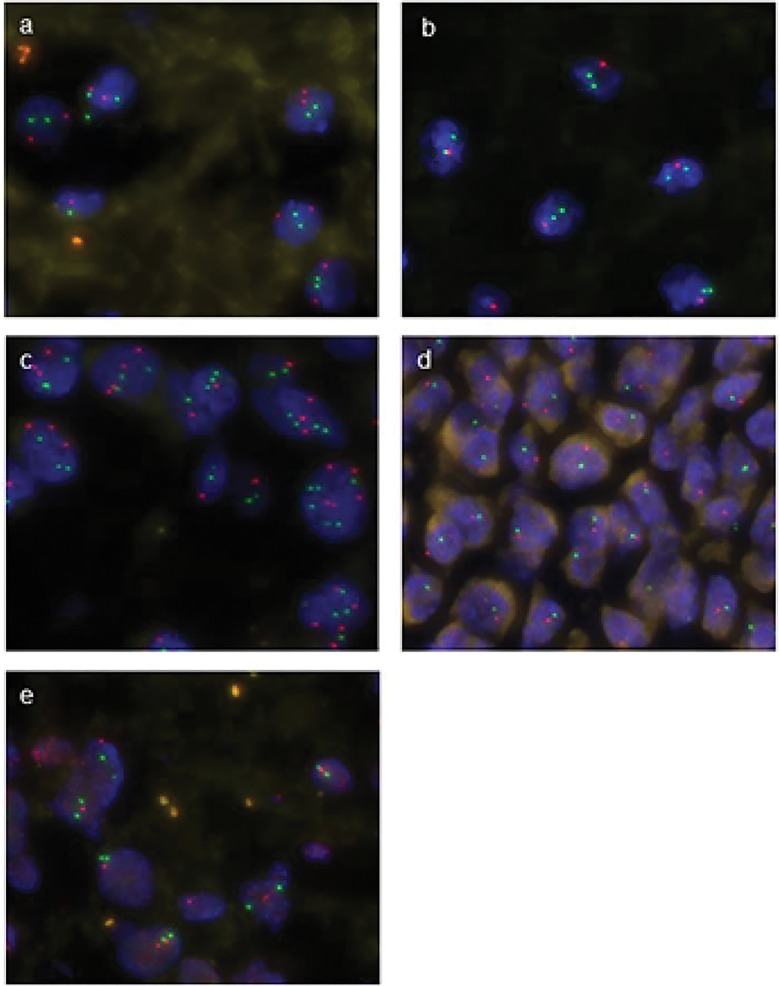
**Representative hybridizations from oligodendrogliomas (a-d) and glioblastoma (e).** a: 9 p normal status with 2 Green control and 2 Red target signals (2R/2G) in majority of tumoral cells. b: 9p deletion with 1R/2G signal in most nuclei. c: 9p imbalance with an increased copy number of red and green signal. d: codeletion of 9p (red) and 9q (green) consistent with a whole chromosome 9 deletion (monosomy 9). e: deletion of PTEN 1R/2G consistent with 10q deletion. Note the presence of frequent background fluorescent artifacts.

Automated analysis of 9p and 9q was successful in the majority of the cases. Only 2 cases labelled with the 9p probe and 3 cases labelled with the 9q probe were not interpretable due to a weak fluorescent signal. In the other cases, the signal was clear and strong enough to classify each cell into to one of 3 groups: normal chromosome 9 status (2 Red target /2 Green control), chromosome 9 deletion (nR/nG ≤ 0.5) and imbalanced chromosome 9 status (nR/nG > 0.5 with n≥3): Fig 1A, 1B and 1C respectively.

In two cases a majority of cells had a 1R/1G combination, which in the specific case of chromosome 9 has been interpreted as a whole chromosome deletion, as described above (Fig 1D). The concordance between manual and automated FISH analysis for chromosome 9 was satisfactory for 25 out of 33 cases (76%; ƙ = 0.65). When we excluded the 2 cases with weak signals for both 9p and 9q, this concordance increased to 25 / 31 cases (81%; ƙ = 0.69). Seven OIIIs showed a 9p deletion, two of which were also deleted for 9q, whereas no 9p deletion was observed in OII cohort (p = 0.002). In AS cohort, all GBMs were deleted for 9p but none of the AII or AIII showed this alteration (p = 0.001). A few cases of 9p imbalance were noted in OII (2/13 = 15%), OIII (1/10 = 10%), AII (2/3 = 67%) and AIII (2/3 = 67%) but no GBMs showed this pattern. There was no significant difference in the distribution of 9p imbalance between OII and OIII, between OG and AS, or between AII and AIII+GBM (Table 1). In our series there was no case with 9q deletion alone.

#### 10q FISH analysis

10q analysis showed a diffuse fluorescent background artefact but the majority of the cases were nevertheless interpretable. Three cases were not interpretable. Two other cases had very weak green fluorescence resulting in an insufficient number of cells detected by automated analysis (less than 50 cells). In the other cases, the automated analysis was uncomplicated and rapid with a moderately satisfactory concordance between manual and automated FISH analysis in 23 cases over 33 cases (70%; ƙ = 0.4). The exclusion of the 5 cases with weak signal improved this concordance to 22 / 28 cases (79%; ƙ = 0.62). In the series as a whole, only GBM cohort showed a clear deletion for chromosome 10q in half of the cases (Fig 1E) with a trend for a concordance between this chromosome arm deletion and the GBM status (p = 0.06). The large majority of the remaining cases showed a normal chromosome 10q status (27/33 = 82%). Only four cases showed a 10q imbalance, among which 2 were OII, 1 OIII and 1 AII (15%, 10% and 33% respectively): Table 1.

#### Number of chromosome arms alteration by FISH analysis

An analysis of individual chromosome alterations (deletions or imbalanced status for 1p, 19q, 9p, 9q and/or 10q) showed no statistical correlation with histological type or grade in our series (Table 1). On the other hand, there was a significant increase in the total number of deletions between OII and OIII (p = 0.001), between OG and AS (p<0.0001) and between GBM and other AS (p = 0.004): Table 1.

### Correlation between clinical, histological, molecular data and OS

**Fig 2:**
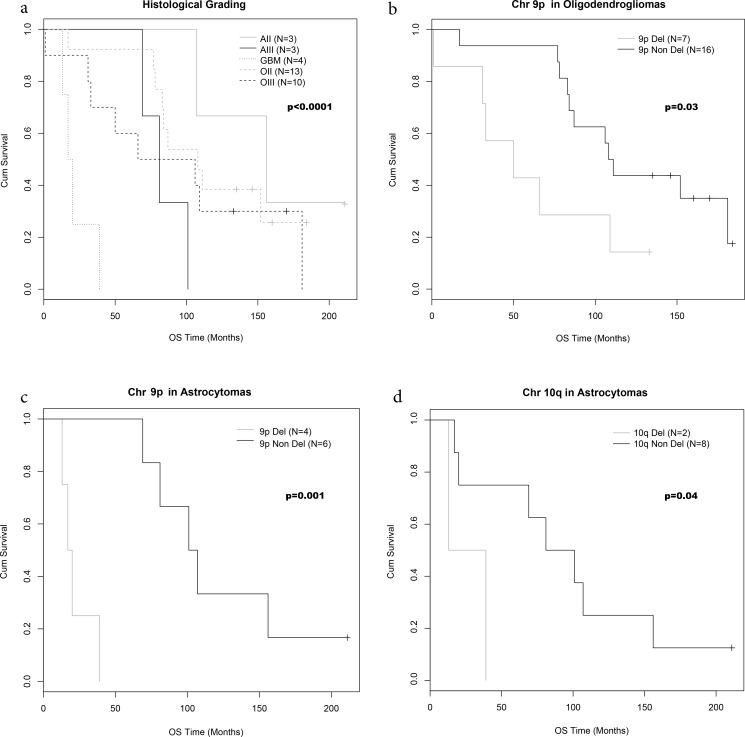
OS curves according to histological and chromosomal status. OS by histological grading (a), chromosome 9p deletion in oligodendrogliomas (b) and astrocytomas (c) and chromosome 10q deletion in astrocytomas.

Histological grading was correlated to OS, with low grade gliomas (OII and AII) showing a better prognosis than high grade gliomas (OIII, AIII and GBM), and a particularly poor prognosis for GBMs: Fig 2A (p<0.001).

The mean OS was higher in OII than in OIII (108 and 86 months respectively) (Table 2) without reaching a statistical prognostic significance (Table 3). Mean OS was 156 months for AII, 81 months for AIII and 19 months for GBMs (Table 2) with a significantly poor prognostic for the latter (p = 0.009): Table 3.

**Table 2:** Correlation to Median OS according to clinical, histological, and molecular data: univariate analysis.

Histological Type		Total cohort	OII	p	OIII	p	AII	p	AIII	p	GBM	p
Median OS (months)		87	108		86		156		81		19	
Recurrence	yes	69	17	-	32	**0,01**	107	-	81	-	/	**- **
	no	107	110		108		184		/		19	
Age at diagnosis	< 50 years	101	87	NS	109	**0,03**	132	-	85	-	20	**- **
	≥ 50 years	82	123		31		211		81		13	
Sex	Male	82	110	NS	83	NS	/	-	75	-	26	**-**
	Female	101	87		86		156		101		19	
Extent of surgery	Biopsy (%)	78	107	NS	/	-	/	-	/	-	13	**- **
	Surgery (%)	94	108		86		156		81		20	
Localization	Frontal	106	99	NS	120	NS	156	-	85	-	15	**- **
	Temporal	77	104		71		/		/		30	
	Parietal	66	/		58		/		81		/	
Postoperative treatment (%)	None	39	83	NS	33	**0,03 **	/	-	/	-	39	**- **
	Radiotherapy	135	135		78		184		/		/	
	Chemotherapy	78	112		92		/		81		17	
	Radio + chemotherapy	108	108		121		107		85		/	
MVP(%)	endocrinoid	108	108	-	86	-	156	-	81	-	13	**-**
	glomeruloid	66	/		/		/		/		20	
Calcifications (%)	Yes	134	152	**0,03**	133	**0,01**	/	-	91	-	/	**- **
	No	77	86		33		156		69		19	
Mitoses /10 HPF	< 5	97	108	-	100	NS	156	-	75	-	15	NS
	≥ 5	50	/		78		/		101		30	
Mib1 (%)	< 12	86	87	NS	67	NS	156	-	81	-	/	**-**
	≥ 12	91	129		78		/		/		19	
INA (%)	< 10	101	153	NS	67	NS	156	-	81	-	20	**-**
	≥ 10	86	87		86		/		/		17	
IDH132H (%)	negative	30	146	NS	/	-	/	-	/	-	20	**- **
	positive	94	98		86		156		81		13	
ATRX (%)	< 10	75	/	-	/	-	107	-	75	-	13	**- **
	≥ 10	94	108		86		184		101		20	
Chr 1p	loss	104	108	-	86	-	211	-	101	-	17	**-**
	no deletion	39	/		/		/		69		30	
	imbalance	94	/		/		132		81		13	
Chr 19q	deletion	106	108	-	86	-	/	-	/	-	/	**-**
	no deletion	30	/		/		/		69		20	
	imbalance	104	/		/		156		91		13	
Chr 9p	deletion	33	/	NS	50	**0,0006**	/	-	/	-	19	**-**
	no deletion	108	87		176		184		85		/	
	imbalance	107	148		106		107		81		/	
Chr 9q	deletion	80	/	-	80	NS	/	-	/	-	/	**-**
	no deletion	87	108		86		156		81		19	
Chr 10q	deletion	26	/	NS	/	-	/	-	/	-	26	NS
	no deletion	84	87		66		159		156		19	
	imbalance	168	148		181		156		/		/	
Chr arm alteration (Mean)	≤ 2	86	87	NS	170	-	211	-	85	-	20	**-**
	> 2	106	148		66		132		81		13	
Chr arm deletion (Mean)	≤ 2	104	108	-	170	**0,02**	156	-	81	-	19	**-**
	> 2	50	/		50		/		/		/	

Statistically significant: p<0.05, NS: Not significant,—: Not applicable

**Table 3:** Correlations between clinical, histological and molecular data and OS: univariate analysis.

		Total cohort		Oligodendrogliomas		Astrocytomas	
		Hazard ratio(95% CI)	p-value	Hazard ratio(95% CI)	p-value	Hazard ratio(95% CI)	p-value
Histological grade	Low / High grade	0.43	**0,03**	0,69	NS	0	**0,009**
Recurrence	No / Yes	2.95	**0,02**	29,35	**0,00002**	0,99	NS
Age at diagnosis	< 50 / ≥ 50 years	1.26	NS	2,02	NS	0,6	NS
Sex	Male / Female	0.95	NS	0,71	NS	3,95	NS
Localization	Frontal / Other	2.08	NS	2	NS	2,7	NS
Postoperative treatment	No / Yes	0.43	NS	0,36	**0,05**	-	-
MVP	No / Yes	1.85	NS	1,45	NS	0.7	**0,05**
Calcifications	No / Yes	0.33	**0,01**	0,16	**0,002**	1,04	NS
Mitoses /10 HPF	< 5 / ≥ 5	1.93	NS	1,88	NS	2,04	NS
Mib1	< 12 / ≥ 12	1.3	NS	1,15	NS	1,53	NS
INA	Negative / Positive	1.22	NS	3,96	NS	-	-
IDH 132H	Negative / Positive	1.89	NS	-	-	9,48	**0,02**
ATRX	Negative / Positive	0.42	NS	-	-	0,6	NS
Chr 1p	deletion / other	2.19	NS	-	-	2	NS
Chr 9p	deletion / other	0.25	**0,0007**	0,34	**0,03**	0	**0,001**
Chr 10q	deletion / other	0.13	**0,003**	-	-	0,16	**0,04**
Chr arm alteration (Mean)	≤ 2 / > 2	1.18	NS	1,29	NS	0,87	NS
Chr arm deletion (Mean)	≤ 2 / > 2	2.09	NS	2,98	**0,03**	-	-

NS: Not significant,—: Not applicable

Age at diagnosis over 50 years was correlated with a shorter OS in OIII (p = 0.03). Tumor recurrence was more frequent in OGs (p = 0.00002) and especially in OIII (p = 0.01): Tables 2 and 3. There were no correlation between age at diagnosis, sex, frontal versus non-frontal tumor location, and OS in OGs and ASs (Tables 2 and 3). Radiochemotherapy showed a better prognosis than radiotherapy or chemotherapy alone or absence of treatment in OGs (p = 0.05) especially in OIII (p = 0.03): Table 2.

Tumor calcifications were strongly correlated with a good OS in the whole OG cohort but not in AS (p = 0.002). This was true for both the OII and OIII cohorts (p = 0.03 and p = 0.01 respectively). MVP was correlated with shorter OS in AS (p = 0.02) but not in OG. There was no significant correlation between high mitotic index or MIB-1 proliferative index and OS in either OG or AS (Tables 2 and 3).

INA overexpression showed no prognostic significance in OG. ATRX mutation showed no prognostic significance in AS. IDH1 wild type was correlated with a poor prognosis in AS (p = 0.02): Table 3.

Chromosome 1p deletion, observed in all OGs, was also observed in one AII and one AIII, both of which showed long OS (211 and 101 months respectively) compared to the median survival of the AII and AIII cohort (156 and 81 months respectively). 1p deletion was also observed in one GBM with a short OS of 17 months (Table 2). Preservation and/or imbalance of 1p and/or 19q showed no correlation with poorer prognosis in either OGs or ASs (Table 2).

Chromosome arm 9p deletion and whole chromosome 9 deletion appeared strongly linked to a poor survival in OG (p = 0.03) and AS (p = 0.001): Fig 2B and 2C. Indeed 9p deletion was predictive of poor OS in both OIII (median OS = 50 months for 9p deleted cases versus 110 months for 9p non-deleted cases, p = 0.03) and in high grade AS (median OS = 19 months for 9p deleted cases versus 104 months for 9p non-deleted cases, p = 0.001).

Deletion of chromosome arm 10q was linked to a poor survival in AS (median OS = 26 months for 10q deleted cases versus 91 months for 10q non-deleted cases, p = 0.04): Fig 2D. Chromosome 9p and 10q imbalance did not appear correlated to histological grade or OS: Table 2.

Alterations on individual chromosome arms 1p, 19q, 9p, 9q and 10q did not appear correlated to prognosis in our series for either OG or AS, whereas the total number of chromosome arms deletions showed prognostic value in OIII (median OS = 50 months for cases with more than 2 deletions versus 170 months for cases with only 2 deletions, p = 0.03): Table 3.

In a multivariate analysis of clinical, histological and molecular data, only chromosome 9p deletion, presence of intratumoral calcifications and tumor recurrence remained independent predictors of poor prognosis, associated with a shorter OS (p = 0.0001, p = 0.002 and p = 0.02 respectively).

## Discussion

Since the 2014 ISN-Haarlem classification and the WHO 2016 Classification of Tumors of the Central Nervous System [1], most tumors previously characterized as oligodendrogliomas based on conventional histologic features are now classified into OGs with the characteristic double molecular signature of IDH mutation and 1p/19q codeletion, or into ASs, the latter including 4 potential subgroups:

- Diffuse astrocytoma, IDH-mutant, often with ATRX loss,
- Diffuse astrocytoma, IDH-wildtype
- Glioblastoma, IDH-mutant
- Glioblastoma, IDH-wildtype

Since this new classification leads to a significant number of revised diagnoses, the pertinence of many previously used diagnostic and prognostic markers has been called into question. All retrospective studies like ours which include a series of diffuse gliomas originally diagnosed as oligodendroglial tumors now require a histological review combined with molecular analysis. This is particularly true for tumors formerly characterized as mixed gliomas of oligoastrocytic lineage (OAs), a concept that most authors now agree should be abandoned [1,2]. In our series, the majority of cases originally diagnosed as OAs were reclassified into AS (6/9) the other cases being reclassified into OGs. Four cases initially diagnosed as OGs were also reclassified into AS, two of them harboring a 1p deletion without loss of 19q, and the two others having no deletion for either 1p or 19q. This degree of reclassification based on modern molecular criteria is similar to that described by others [2].

Once our cases were reclassified there was no clinical difference between OII and OIII regarding sex, age at diagnosis or extent of surgery, similar to findings in previous studies [19,20]. The majority of OGs were located in the frontal lobe as expected, but there was no significant association between temporal lobe location and maintenance of 1p/19q status as has been described by some authors [21,22]. This may be explained by the small size of our AS cohort.

MVP as defined by the 2007 WHO Classification of Tumors of the Central Nervous System [23] was present only in OIII and GBM. The presence of MVP in OGs was not correlated to OS, unlike that reported by some authors [10,24,25]. In ASs the presence of MVP was sufficient for a diagnosis of GBM [23] thus a correlation with poor prognosis was not surprising. Mitotic index and Mib1 proliferative index were higher, as expected, in high grade OG and AS compared to their lower grade counterparts [23,24] but did not otherwise affect OS. Intratumoral calcifications were observed in both OG and AS as well-described in the literature [10,23] and correlated with a better OS in univariate and multivariate analysis. Very few studies report the link between calcification and a better prognosis in gliomas [26,27] and it has been suggested that calcification might be a consequence of the slow growth and indolent course of a less-aggressive tumor [28].

In our series INA expression was strongly correlated with 1p/19q codeletion and thus serves as an immunohistochemical marker of OG status as described in the literature [10,13], but we were unable to show a correlation with INA expression and OS as reported previously. This may be a consequence of the smaller number of cases examined in our study (33 patients versus 203 and 92 respectively). ATRX mutation was determined by the loss of protein expression in tumor cells as observed by immunohistochemistry [16]. As expected, all OGs and the majority of GBMs (75%) were ATRX positive (non-mutated) [1]. The majority of our AIII (66%) appeared ATRX mutated as expected in the literature (57% versus 73%). Only 33% of our AII appeared mutated for ATRX. This represents a smaller proportion of cases than usually described in the literature (45% versus 67%) [16,29,30], but may be a consequence of the small size of our AII cohort. In our AS cohort as a whole, ATRX mutation appeared closely associated with IDH mutation (100% concordance) as described in the literature [30]. All our OGs and AII and AIII were IDH1/2 mutated as expected by the 2016 WHO definition of OGs and as reported in the literature for the majority of AS [31,32]. Despite the small size of our cohort, we found this mutation was correlated with a better prognosis in the ASs as expected from the literature [31,32]. The single IDH-mutant GBM in our series showed no better outcome than the three other IDH-wildtype GBMs.

In this study, automated analysis of chromosomes 1p and 19q was uncomplicated to perform except for a few of the oldest cases, corresponding mostly to those from years 1998 and 1999, for which problems in processing and long-term storage may be responsible. All OGs were 1p and 19q codeleted as defined by the WHO. Deletion of 1p alone was also observed in few cases of ASs (3 /10 cases). In our ASs cohort, 1p deletion was correlated with a better prognosis in AII (211 months versus 131 for the mean OS cohort) and AIII (101 months versus 75) but not in GBMs (17 months versus 24) in accordance with the literature [33]. Since some studies reported a strong correlation between 1p and/or 19q polysomy and shorter OS in oligodendroglial tumors [14,15,34] determination of the percentage of tumor cells polysomic for 1p and 19q is routinely performed in our institution. Polysomy as defined by these authors is taken into account by our algorithm and assigned to the imbalanced status subgroup, as described previously [12]. In the present work, all cases with high percentage of tumour cells showing imbalanced 1p and/or 19q status belonged to the AS cohort but this status was not necessarily predictive of a high grade or a poor prognosis. The total absence of imbalanced cases in our OG cohort is a consequence of the 2016 WHO criteria whereas previous studies which reported occasional OGs with imbalanced chromosome 1p/19q status are in retrospect probably inhomogeneous, with no reference to IDH1/2 status, and often including the now discredited diagnosis of oligoastrocytoma. Many of their cases would nowadays be considered as astrocytic tumors. In order to further characterize our series we performed additional molecular testing which revealed that of the 10 reclassified AS, 4 were ATRX mutated which is uncommon in IDH-mutant tumors with 1p/19q codeletion [30,35], 3 others were IDH wild type which precludes a definition of OG [1], 2 other cases presented a 1p deletion alone which also precludes the WHO definition of OGs and the final reclassified case included fewer than 20% of cells with 1p and 19q deletions amidst a large majority of cells with imbalanced signals, which did not fit with our previously established deletion criteria. These criteria, which set a stringent level (at least > 55% of tumor cells) for the definition of the 1p/19q codeletion by FISH, identified all IDH1/2 mutated and ATRX wild type gliomas that meet the 2016 WHO Criteria for OGs.

Chromosome arm 9p deletion is a common progression-associated alteration in high grade and/or recurrent OG [9,36] and in high grade AS [35]. This deletion has been correlated to necrosis and MVP in OG [10,11,37] and to unfavourable outcome in both low grade gliomas [38] and anaplastic gliomas. These effects are attributed to the loss of the *CDKN2A* locus on chromosome 9p21 which includes the genes for p16^INK4A^ and p14^ARF^. As expected, our results confirmed the strong prognostic value of 9p status in both high grade OGs and ASs which was still present in multivariate analysis. Our study also highlighted the value of automated analysis of chromosome 9p by FISH, which was easy to perform and showed a good concordance with the manual control analysis (κ = 0.69). Our choice of a green control located on chromosome 9q instead of a centromeric control as usually chosen in the FISH literature on chromosome 9p [9,35] allowed us to detect the rare cases of OG with concomitant 9q deletion as previously described in OGs by Comparative Genomic Hybridization array studies [8,10]. Our results underline the value of studying chromosome 9 status in all gliomas. We recommend that chromosome 9 should be investigated at the same time as chromosomes 1p and 19q because its deletion could justify reclassification of gliomas into a higher grade, especially in cases where little tumor material is available for study and/or not very representative of the whole tumor.

Chromosome arm 10q deletion has long been known to be common in high grade gliomas [39] and especially to GBMs [40,41]. Several tumor suppressors including PTEN have been identified on this arm. Chromosome arm 10q deletion has also been described in OGs, with some authors reporting that 10% to 35% of OGs harbor this deletion [37,42–44] but not others [9,10]. Chromosome arm 10q deletion in OGs has been associated with a worse prognosis [42,43]. Once again these studies need to be re-evaluated in the light of the 2016 WHO Classification. In our study 10q deletion was present in 50% of our GBM cohort in concordance with the literature [40] and associated to a poor OS even for the case with a concomitant presence of 1p deletion highlighting the pejorative nature of 10q deletion. Chromosome arm 10q deletion was not observed in any of our OGs cases. A careful study of the past literature reveals that the presence of a whole 10q arm loss in codeleted 1p/19q OGs is very infrequent (0 to 6% of cases) [8–10,45–47] suggesting that this event is more related to astrocytic differentiation. Those studies reporting a high rate of 10q deletion associated to 1p/19p codeletion assessed Loss Of Heterozygosity after taking into consideration both 10q focal mutations or whole arm deletion [37,42,44] or used a FISH technique [43] in which the percentage rate defining the 1p/19q codeletion status (20%) was much lower than the 55% cut-off used in our study. We think it likely that some OGs as defined by these techniques would be better included with the AS group. Based on our study, automated FISH analysis is a reliable technique to study chromosome 10 but performing a systematic 10q status study in all gliomas seems to us excessive, since 10q deletion is rarely present except in GBMs. In our experience 10q deletion analysis can be helpful for the diagnosis of difficult cases (small biopsies, hemorrhagic samples, peri-tumoral samples) for which histologic analysis alone is not sufficient. In these cases the presence of 10q deletion could justify the diagnosis of a high grade AS, probably a GBM.

In gliomas as in majority of solid tumors, almost all chromosomes are more frequently lost than gained [48]. The trend to lose chromosomes is observed both in low and high grade gliomas with an increasing number of aneuploid cells and chromosomes losses seen in higher grades [48]. The number of whole chromosome aberrations per tumor increases during cancer progression. The total number of chromosome arm alterations appears higher in ASs than OGs and is correlated to a shorter OS for both high grade AS and OG [10]. Unlike whole genome exploration techniques such as Comparative Genomic Hybridization which allows the simultaneous analysis of all chromosome arms, our present study focused only 8 different chromosome arms (1p, 9p, 10q and 19q and their corresponding opposite arms used as control) which could explain the absence of statistical significance in our cohort when comparing the mean number of chromosome alterations and OS. The mean number of chromosome arm deletions was correlated to OS in our OG cohort but provides no additional prognosis information over the study of 9p deletion; therefore we see no basis to recommend that the mean number of chromosome arm deletions be calculated in routine practice, at least with our 4 chromosomes panel.

## Conclusions

This study is, to the best of our knowledge, the first published attempt to investigate the feasibility and reliability of automated 1p, 19q, 9p and 10q FISH analysis and its value in the diagnosis and the prognosis of oligodendroglial tumors. Automated determination of 1p/19q codeletion allowed us to easily reclassify our cohort into OGs and ASs with evidence of a better response to the radiochemotherapy treatment for OGs. 1p and/or 19q imbalanced status determination appeared important, not for a prognosis purpose as previously reported in the literature, but as a diagnostic tool, all the imbalanced cases in our series being classified into ASs. 9p deletion appeared strongly correlated to high grade OGs and ASs and to a poor OS in both univariate and multivariate analysis which was not well recognized in the past literature, especially for ASs. However, our results, although statistically significant, were observed on a small mono-institutional cohort and the potential role of 9p deletion in the management of gliomas should be confirmed and clarified in further clinical studies and on different FISH platforms. Very few 10q deleted cases were observed in our series, all of them in GBMs with a correspondingly poor OS. After applying stringent criteria in the definition of 1p/19q status we found no case of 10q deletion in OGs cohort. Its systematic study in the management of these gliomas is a matter of debate and should be proposed only in specific cases.

Given the growing importance of molecular characterization in the diagnosis and the management of gliomas, as recommended in the 2016 WHO Classification of Tumours of the Central Nervous System, and given the easy availability of the FISH technique in most pathology laboratories, our work highlights the utility of automated FISH in the determination of 1p, 9p, 10q and 19q status. Our work can easily be reproduced in other institutions since all commercial FISH platforms are equipped with automated analysis software similar to that which we used. Automated analysis covers more cells in less time than a manual analysis, with consequent improvements in turnaround time and decreased technical costs.

## Supporting Information

S1 File Research Ethics Committee opinion. (PDF)
