# Supplementary material for: Contribution of 1p, 19q, 9p and 10q Automated Analysis by FISH to the Diagnosis and Prognosis of Oligodendroglial Tumors According to WHO 2016 Guidelines
Source: PLoS One. 2016 Dec 28;11(12):e0168728. doi: 10.1371/journal.pone.0168728 (PMC5193469; doi:10.1371/journal.pone.0168728)
Supplement: S1 File — (PDF) [file pone.0168728.s001.pdf]

Par courrier électronique seulement

Le 21 septembre 2015

Docteur Stéphan Saikali
L'HDQ du CHU de Québec
Local 3370

Objet : Réponse à la demande d'avis 2016-2638
Intérêt de la caractérisation moléculaire des anomalies chromosomiques 1p, 19q, 9p, 9q, et 10q dans la prise en charge des oligodendrogliomes

Docteur,

Votre demande datée du 25 août 2015 concernant la nécessité ou non d'obtenir une approbation du Comité d'éthique de la recherche a été évaluée. Après l'avoir analysé, il appert que, selon l'Énoncé de politique des trois Conseils, *Éthique de la recherche avec des êtres humains, 2014 (EPTC2, article 2,5),* votre devis de recherche n'est pas considéré comme un projet de recherche nécessitant l'approbation d'un comité d'éthique de la recherche.

Conséquemment le CÉR du CHU de Québec n'a pas à approuver votre projet afin qu'il puisse se dérouler au sein de l'établissement. Toutefois, les considérations habituelles pour l'accès aux dossiers et le respect de la confidentialité selon les règles des Bonnes Pratiques Cliniques sont applicables.

Les informations recueillies ne devront servir qu'aux seuls objectifs de la présente activité d'évaluation et ne devront pas faire l'objet d'une banque de données ou être utilisées ultérieurement à d'autres fins. De plus, aucun contact avec les patients ou les parents (dans le cas de personnes mineures) ne sera effectué dans le cadre de cette activité d'évaluation. Il est recommandé de procéder à l'anonymisation irréversible une fois que les analyses complétées afin de permettre une protection maximale de la confidentialité.

Je vous prie d'agréer, Docteur, l'expression de mes sentiments les meilleurs.

Ana Marin, Ph.D.
Comité d'éthique de la recherche
CHU de Québec – Université Laval

AM/ad
